# Trends and Inequalities in Use of Maternal Health Care Services in Nepal: Strategy in the Search for Improvements

**DOI:** 10.1155/2017/5079234

**Published:** 2017-07-20

**Authors:** Suresh Mehata, Yuba Raj Paudel, Maureen Dariang, Krishna Kumar Aryal, Bibek Kumar Lal, Mukti Nath Khanal, Deborah Thomas

**Affiliations:** ^1^Nepal Public Health Foundation, Kathmandu 44600, Nepal; ^2^Nepal Health Sector Support Program, Ministry of Health, Kathmandu 44600, Nepal; ^3^Nepal Health Research Council, Ministry of Health, Kathmandu 44600, Nepal; ^4^Ministry of Health, Kathmandu 44600, Nepal

## Abstract

**Background:**

Nepal has made significant progress against the Millennium Development Goals for maternal and child health over the past two decades. However, disparities in use of maternal health services persist along geographic, economic, and sociocultural lines.

**Methods:**

Trends and inequalities in the use of maternal health services in Nepal between 1994 and 2011 were examined using four Nepal Demographic and Health Surveys (NDHS), nationally representative cross-sectional surveys conducted by interviewing women who gave birth 3–5 years prior to the survey. Sociodemographic disparities in maternal health service utilization were measured. Rate difference, rate ratios, and concentration index were calculated to measure income inequalities.

**Findings:**

The percentage of mothers that received four antenatal care (ANC) consultations increased from 9% to 54%, the institutional delivery rate increased from 6% to 47%, and the cesarean section (C-section) rate increased from 1% in 1994 to 6% in 2011. The ratio of the richest and the poorest quintile mothers for use of four ANC, institutional delivery, and C-section delivery were 5.08 (95% CI: 3.82–6.76), 9.00 (95% CI: 6.55–12.37), and 9.37 (95% CI: 4.22–20.83), respectively. However, inequality is reducing over time; for the use of four ANC services, the concentration index fell from 0.60 (95% CI: 0.56–0.64) in 1994–1996 to 0.31 (95% CI: 0.29–0.33) in 2009–2011. For institutional delivery, the concentration index fell from 0.65 (95% CI: 0.62–0.70) to 0.40 (95% CI: 0.38–0.40) between 1994–1996 and 2009–2011. For C-section deliveries, an increase in concentration index was observed, 0.64 (95% CI: 0.51–0.77); 0.76 (95% CI: 0.64–0.88); 0.77 (95% CI: 0.71–0.84); and 0.66 (95% CI: 0.60–0.72) in the periods 1994–1996, 1999–2001, 2004–2006, and 2009–2011, respectively. All sociodemographic variables were significant predictors of use of maternal health services, out of which maternal education was the most powerful.

**Conclusion:**

To increase equitable use of maternal health services in Nepal there is a need to strengthen the health system to increase access to and utilization of services among poorer women, those with less education, and those living in remote areas. Beyond the health sector stronger efforts are needed to tackle the root causes of health inequality, reduce poverty, increase female education, eradicate caste/ethnicity based social discrimination, and invest in the development of remote areas.

## 1. Introduction

In many developing countries, wide inequalities in use of health services intensify disparities in the health outcomes of their people [1–6]. Enhancing equitable access to and utilization of health services is crucial to achieving faster and more sustainable improvements in health status [4].

Nepal has made significant progress in meeting maternal and child health related Millennium Development Goals and has achieved remarkable reductions in maternal, newborn, infant, and under-five mortality over the past two decades [7]. However, stark disparities in utilization of services and health outcomes persist along geographic, economic, and sociocultural lines [8–11]. The Nepal Maternal Mortality and Morbidity Study (2008) which was undertaken in eight districts found maternal mortality ratio varied by geographical area with higher rates in mountain districts compared to those in the hills and plains (otherwise known as Terai in Nepal) [12]. Likewise, studies have revealed disparities in access to and use of maternal health services by caste and ethnicity, rural-urban residence, and economic status. Low use of maternal health services has been observed among Dalits (the castes who were formerly considered “untouchable” according to the Hindu varna system), Muslims, and Terai/Madhesi (the Terai/Madhesi people are native inhabitants of the flat southern region of Nepal) peoples. The use of maternal health care has been found to be low among those who reside in rural areas and the poor [10, 11, 13, 14].

A review of policies and programmes reveal that Nepal has largely taken an undifferentiated approach to delivering maternal and child health (MCH) services [15]. While MCH services are a health sector priority in Nepal, attention has been focused on achieving population-based targets with programmes and interventions tested in easily accessible areas and without due consideration to the social determinants that affect access to services. In the pursuit of national targets less attention has been given to equitable access and reaching the most disadvantaged who face greater geographical, sociocultural, and economic barriers to accessing care. In other words, efforts to improve national level maternal health indicators until now have often overlooked subnational and socioeconomic inequalities [16]. It is therefore essential that the social determinants of service use, and the extent and nature of disparities are examined and understood, so that strategies and programmes to address inequities can be developed.

The objectives of this study were to assess the social determinants of inequalities in use of maternal health services in Nepal by drawing on national household surveys over an 18-year period from 1994 to 2011.

## 2. Materials and Methods

### 2.1. Conceptual Framework

The social determinants of health are factors contributing to the inequitable distribution of health status across social groups. These determinants align with social position, which is interlinked with a complex web of societal and cultural mechanisms that generate and uphold social hierarchies. They impact health status through material circumstances, psychosocial circumstances, and behavioural and/or biological factors. Improvements in public health therefore depend not only on access to health care but also on addressing a range of other social determinants of health [17]. On the basis of Nepal's socioeconomic, political, cultural, and ecological context, and available evidence on factors that affect the use of health care in Nepal [17], the social determinants selected for this study are mothers' education, caste and ethnicity, wealth, rural-urban residence, and ecological zones.

### 2.2. Data Source

Data were obtained from four nationally representative cross-sectional surveys conducted in Nepal in 1996 (Nepal Family Health Survey, NFHS), 2001, 2006, and 2011 (Nepal Demographic and Health Surveys, NDHS). These surveys provide data on reproductive health including maternal health care practices across time. The surveys were based on two-stage, systematic cluster random sampling; sample size and response rates are presented in Table 1.

The datasets were downloaded with permission from Measure DHS website [18]. Variables from household-level files were merged into the child-level files by using household and case identification variables for matching. For 1996 and 2001 an additional step was taken to merge data from separate wealth index files into household-level files prior to merging into the child-level files. Child-level files were used for this analysis. (Table 1). A sample of 22,437 women who had given birth between 1994 and 2011 were derived from 4 surveys to assess sociodemographic characteristics and perform trend analysis. Since NFHS 1996 collected data for a period of three years before the survey, data from subsequent DHS surveys were also restricted to a three-year reference period while analyzing determinants of service use for uniformity of the data period.

### 2.3. Derivation of Variables

The dependent variables in this analysis were use of antenatal care, institutional delivery, and delivery by cesarean section. The indicators analyzed in this study are defined in Table 2.

Independent variables included in this study were mothers' education, caste and ethnicity, wealth quintile, rural-urban residence, and ecological zone.

### 2.4. Data Analysis

All analyses were first performed using the full national sample. Data was then disaggregated by mothers' education (no education, primary, secondary, and higher education), wealth quintiles, caste and ethnicity (Brahmin/Chhetri, Terai/Madhesi Other Caste, Dalit, Newar, Janajati, Muslim, and Other), rural-urban residence, and ecological zone (mountain, hill, and Terai), to assess disparities in use of maternal health services. All analyses were conducted using STATA 13. Prevalence values reported were weighted by sample weights to provide population estimates.

#### 2.4.1. Measurement of Trend

Bivariate and multivariate logistic regression was used to assess the annual rate of change (ARC) in service use (e.g., 4 ANC, institutional birth) [19].

#### 2.4.2. Measurement of Income Inequality

Rate difference and rate ratios were calculated to measure income inequalities [20, 21]. However, rate difference and rate ratios only take into account the two extreme socioeconomic groups; for example, in wealth quintiles, only first and fifth are taken into account. The wealth quintiles in the middle, that is, second, third, and fourth, are disregarded. Hence rate difference and rate ratios do not give a composite measure of inequality [22]. To address this limitation, concentration index and 95% of confidence interval (95% CI) were also used in order to assess income inequality over time. The concentration index takes on values between −1 (indicates health care utilization is concentrated among the poor) and +1 (indicates health care utilization is concentrated among the rich).

#### 2.4.3. Measurement of Social Determinants of Inequality

Binary and multivariate logistic regression was performed to assess inequalities in use of health services by social determinant.

#### 2.4.4. Ethics

Ethical approval was obtained from the Nepal Health Research Council (NHRC) ethical review committee for all rounds of Demographic Health Surveys (DHS). Furthermore, before starting an interview, enumerators informed all of the respondents of the purpose of the survey; showed authorization letters from the Ministry of Health (MoH); and informed respondents that they were under no obligation to participate in the survey and that if they did choose to participate, all responses would remain confidential. The enumerators subsequently requested verbal consent from the respondents to begin the interview as per NHRC ethical review guidelines.

## 3. Results

### 3.1. Trends in Sociodemographic Characteristics of the Population

Data from the four NDHS surveys clearly showed that there have been remarkable sociodemographic changes in Nepal over the last two decades. The proportion of women with no education among those who delivered in last five years decreased from 79% in 1996 to 47% in 2011. The proportion of respondents in urban areas increased from 6% in 1996 to 9% in 2011. (Table 3).

### 3.2. Trends in Use of Maternal Health Care Services

**Figure 1:**
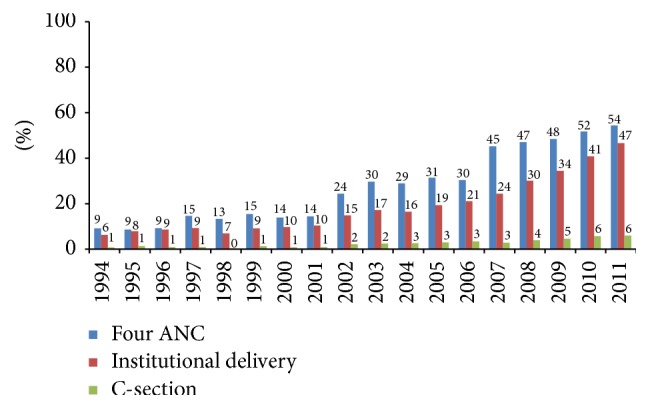
Trend in use of four ANC, institutional delivery, and C-section  1994–2011.

As shown in Figure 1, the percentage of mothers with four antenatal care (ANC) visits increased from 9% to 54% and the institutional delivery rate increased from 6% to 47% between 1994 and 2011. Meanwhile, the C-section rate increased sixfold from 1% in 1994 to 6% in 2011. The rates of annual increase were statistically significant for all three indicators (Table 4). However, the annual rate of increase was more pronounced for use of four ANC and institutional delivery than C-section delivery. Adjusted yearly trend was 18% (AOR: 1.180; 95% CI: 1.153–1.191) for four ANC visits; 17% (AOR: 1.174; 95% CI: 1.154–1.193) for institutional delivery; and 13% (AOR: 1.128; 95% CI: 1.098–1.158) for C-section delivery.

Women who delivered during 2009–2011 were nearly 10 times (AOR: 9.8; 95% CI: 7.65–12.55) more likely to deliver at health facilities and nearly 5 times (AOR: 4.83; 95% CI: 3.13–7.46) more likely to deliver by C-section than women who delivered during 1994–1996 (Table 5).

**Figure 2:**
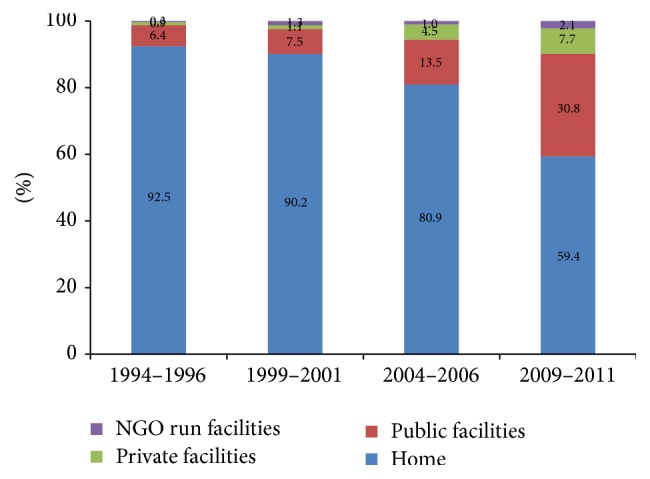
Distribution of place of delivery over time.

Figure 2 illustrates that institutional delivery rate increased both in public and private sector facilities between 1994–1996 and 2009–2011. While public health facilities continue to have a larger number of deliveries in comparison to private, the rate of increase in institutional delivery was higher among for-profit private facilities compared to public facilities. In 1994–1996, only 1% of deliveries were conducted in for-profit private sector health facilities, which increased to 8% in 2009–2011, while proportion of deliveries conducted at public facilities increased from 6% to 31%. Institutional deliveries in NGO facilities increased from 0.3% to 1.2% (Figure 2). Proportion of deliveries conducted at home drastically reduced from 92.5 in 1994–1996 to 59.4 during 2009–2011.

### 3.3. Trends in Inequalities in Utilization of Maternity Care Services

**Figure 3:**
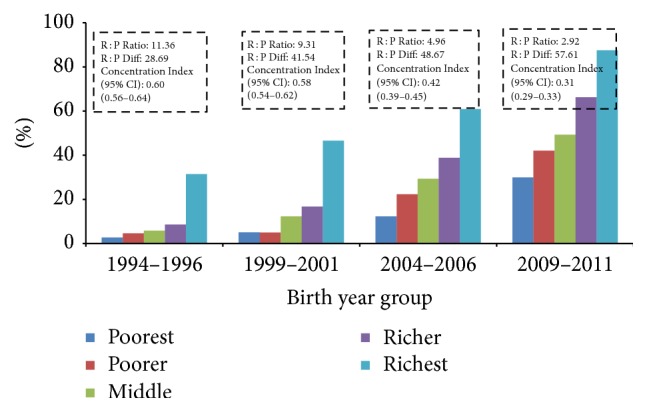
Income inequality in use of four antenatal care visits over time.

**Figure 4:**
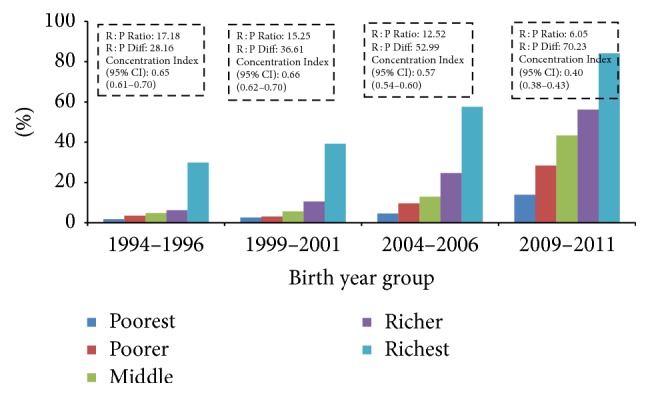
Income inequality in use of facility delivery over time.

**Figure 5:**
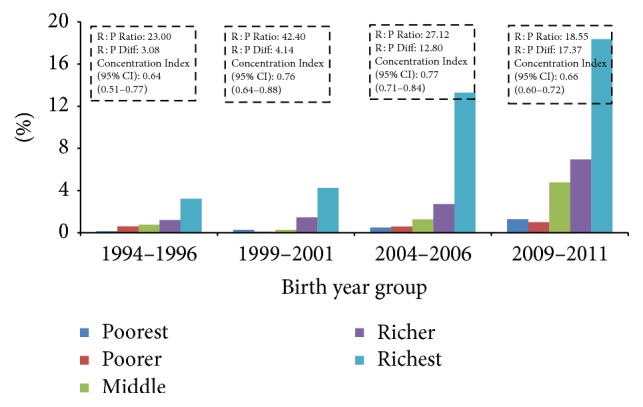
Income inequality in use of C-section over time.

Current analysis found inequality in use of all three maternal health services (4ANC, institutional delivery, and C-section), with inequality greater for C-section delivery than institutional delivery or use of four ANC (Figures 3, 4, and 5 and Table 5). Mothers from the richest wealth quintile were more than five times more likely to have 4ANC consultations (AOR: 5.08, 95% CI: 3.82–6.76), nine times more likely to give birth at health institutions (AOR: 9.00; 95% CI: 6.55–12.37), and nearly 10 times more likely to give birth by C-section (AOR: 9.37; 95% CI: 4.22–20.83) (Table 5). However, the current analysis indicates that inequality is reducing over time in terms of rich : poor ratio and concentration indices (Figures 3, 4, and 5). The rich : poor ratio was reduced from 11.36 in 1994–1996 to 2.92 in 2009–2011 and the concentration index dropped from 0.60 (95% CI: 0.56–0.64) to 0.31 (95% CI: 0.29–0.33) for 4ANC consultations. For institutional delivery, the rich : poor ratio reduced from 17.18 to 6.05 and the concentration index from 0.65 (95% CI: 0.62–0.70) to 0.40 (95% CI: 0.38–0.40) between 1994–1996 and 2009–2011. For C-section deliveries, although the rich : poor ratio decreased from 23.00 to 18.55, an increase in concentration index was observed, 0.64 (95% CI: 0.51–0.77); 0.76 (95% CI: 0.64–0.88); 0.77 (95% CI: 0.71–0.84) between the periods 1994–1996, 1999–2001, and 2004–2006, respectively, and decreased to 0.66 (95% CI: 0.60–0.72) during 2009–2011. However, no equity-gain was observed for the three indicators in terms of rich : poor absolute differences in percent points over the time period (Figures 4 and 5). Table 4 shows that the yearly increase of facility delivery and C-section delivery rate was lower among richest mothers than others although the difference was not statistically significant, and the highest yearly rate of increase was observed among middle quintile mothers. For four ANC use, the yearly increase rate was significantly higher among the poorest women in comparison to richest women (Table 4).

Inequality in maternal health care utilization was present by place of residence as well. Women from urban areas were nearly 1.5 times more likely to receive four ANC (AOR 1.46; 95% CI: 1.19–1.79), nearly three times more likely to have institutional delivery (AOR 2.72; 95% CI: 2.23–3.31), and nearly twice as likely to deliver by C-section (AOR 1.83; 95% CI: 1.29–2.59) in comparison to women from rural areas. However, it is important to note that yearly increase for the use of four ANC was 19% (95% CI: 17.0%–21.2%) in rural areas compared to 12% (95% CI: 8.5%–14.5%) in urban areas. The yearly increase in institutional delivery rate was 19% (95% CI: 16.3%–20.7%) in rural areas compared to 11% (95% CI: 8.4%–14.6%) in urban areas; and for C-section delivery, the adjusted yearly increase was 14% (95% CI: 9.7%–17.4%) in rural areas and 11% (95% CI: 6.7%–15.7%) in urban areas (Table 4). Hence, data suggests inequality in use of maternal health care by rural-urban place of residence is reducing over time.

The highest but insignificant increase in annual trend (adjusted) in use of four ANC 26% (95% CI: 19.9%–32.2%) and institutional delivery 21% (95% CI: 14.3%–27.7%) was observed in mountain residents compared to others. However, a higher increase in C-section rate was observed among Terai 16% (95% CI: 11.4%–20.1%) and hill 9% (95% CI: 4.3%–13.3%) residents compared to mountain residents. Furthermore, there is no significant increase in C-section rate among mountain residents (AOR: 1.046; 95% CI: 0.917–1.194) (Table 4). The odds of delivery by C-section among Terai residents are almost double compared to mountain residents (AOR: 1.90; 95% CI: 1.07–3.38) (Table 5).

In terms of caste/ethnicity, the highest rate of increase in use of four ANC was observed among Janajatis (AOR: 1.218; 95% CI: 1.182–1.255) and Muslims (AOR: 1.208; 95% CI: 1.133–1.228). For institutional delivery the highest rate of increase was among Janajatis (AOR: 1.190; 95% CI: 1.156–1.226); for C-section it was for Muslims (AOR: 1.388; 95% CI: 1.169–64.9). The lowest rate of increase was observed among Newars for four ANC (AOR: 1.108; 95% CI: 1.047–1.172), institutional delivery (AOR: 1.135; 95% CI: 1.057–1.219), and C-section (AOR: 1.030; 95% CI: 0.960–1.105).

### 3.4. Sociodemographic Predictors of Use of Maternity Care Services

All sociodemographic variables were significantly associated with utilization of 4ANC, institutional delivery, and cesarean section both in bivariate and multivariate logistic regression. Among sociodemographic variables, maternal education showed the strongest association with use of 4 ANC. Mothers with higher education were about ten times more likely (AOR: 10.38; 95% CI: 6.81–15.81) to use four ANC compared to women with no education. Wealth index showed the strongest association with use of institutional birth and use of C-section. Women from the highest wealth quintile were 9 times more likely to give birth at a facility (AOR 9.00; 95% CI: 6.55–12.37) and nine times more likely to give birth by C-section (AOR 9.37; 95% CI: 4.22–20.83) in comparison to women from the lowest wealth quintile. Maternal education was the second most powerful predictor for institutional birth and use of C-section. Women with higher education were eight times more likely (AOR: 7.81; 95% CI: 5.08–11.99) to give birth at the facility and almost 3 times more likely (AOR: 3.02; 95% CI: 1.63–5.60) to deliver their babies by C-section (Table 5) in comparison to women with no education. Rural-urban residence was the third most powerful predictor for use of four ANC and institutional delivery and C-section delivery. Urban women were one and half times more likely than rural women to have 4 ANC (AOR: 1.46; 95% CI: 1.19–1.79), almost three times more likely to deliver in an institution (AOR: 2.72; 95% CI: 2.23–3.31), and almost two times more likely to have a C-section (AOR: 1.83; 95% CI: 1.29–2.59).

The use of four ANC and facility delivery services was found to decrease with increasing age. However, delivery by C-section was found to increase with increasing age. Caste and ethnicity were the fourth most powerful predictors of all three outcome variables. The odds of 4ANC (AOR: 2.57; 95% CI: 1.76–3.77), institutional delivery (AOR: 2.17; 95% CI: 1.51–3.13), and C-section delivery (AOR: 2.28; 95% CI: 1.25–4.15) are highest among Newars compared to other caste/ethnic groups when compared to Dalits. The lowest odds of four ANC were observed among Terai Madeshi Castes (AOR: 0.65; 95% CI: 0.49–0.87) and Muslims (AOR: 0.66; 95% CI: 0.47–0.93) compared to Dalits. The lowest odds (but statistically insignificant) of institutional delivery were observed among Janajatis (AOR: 0.85; 95% CI: 0.67–1.08) compared to Dalits, and for C-section this was among Muslims (AOR: 0.72; 95% CI: 0.24–2.16) (Table 5). Ecological zone showed the weakest (but significant) association with use of 4ANC services, institutional delivery, and delivery by C-section. The odds of institutional delivery are 1.48 (95% CI: 1.11–1.98) for Terai residents compared to mountain residents.

## 4. Discussion

In this study, we analyzed the trends, inequalities, and social determinants of the use of maternal health services in Nepal over the last eighteen years. We investigated the association of four ANC, institutional delivery, and C-section with key social determinants of health in Nepal. Findings indicate substantial progress both in increased use and reduced social gradient in use of these health services over the last eighteen years. Despite improvement in income equality in utilization of maternal health services over time as shown by concentration indices, use of maternal health services in Nepal still remain inequitable on many levels. Access to maternal health care especially institutional birth is egregiously low in Nepal overall, and particularly among women with no or lower education, women from low wealth quintile households, disadvantaged caste/ethnic groups, and women from remote areas.

Analysis of data from four DHS surveys indicates that the most significant determinants of inequality in maternal health use are maternal education and wealth index with both showing an apparent dose-response relationship. Women from the poorest wealth quintile and those with no formal education were the most disadvantaged group in terms of their use of maternal health services in Nepal regardless of age, place of residence (rural-urban), ecological zone (mountain, hill and Terai), or caste/ethnicity. The link between maternal education and wealth and utilization of maternal health services has been well documented internationally [23, 24] and these findings further verify the association.

Disparity in coverage of C-section has persisted since 1994–1996 and increased in the two subsequent surveys of 2001 and 2006 and remained constant in 2011. During 1994–1996, coverage of C-section was generally low with high inequality. In recent years, the C-section rate has increased with increased coverage of emergency obstetric care. However, use of C-section was highly concentrated among urban, wealthy, and higher educated women. Given continuing poor access to C-section facilities in hard-to reach areas, further expansion of comprehensive emergency obstetric care is required nationwide especially in underserved areas. Notably the study found no significant increase in the use of C-section among mountain residents. There is a growing concern of irrational use of C-section by women from higher wealth quintile and with higher education in urban areas of Nepal, as has been reported in other countries due to the over-medicalization of child birth, maternal, and provider preferences [25].

There has been a higher rate of increase in institutional deliveries at private facilities, which overwhelmingly attract better off users, compared to public facilities [26]. Although users may prefer private facilities due to shorter waiting time, better responsiveness, and improved confidentiality, many private facilities run unregulated in Nepal and overcharging is often reported with the unit cost of deliveries higher in private than public facilities and quality of care a continuing challenge in the private sector [27]. Moreover, private health facilities are concentrated in Nepal's urban areas where less than one-fifth (17%) of the total population live [28].

Demand side financing was introduced in Nepal in 2009 to remove the financial barriers to institutional deliveries [29]. Experience from the early years of implementation revealed low coverage of the programme [30]. While this has increased over time, the low amount paid to women to help cover their transportation costs leaves many paying out of pocket and poor women and those from mountain areas have been found to be reaping the least benefits [31]. Since coverage of institutional delivery is now above 50%, targeting benefits to poor women and those from remote areas should now be a priority. Studies from Cambodia and Pakistan demonstrated that targeted voucher programs were successful in enabling poor women to access institutional deliveries and reducing use inequalities [32, 33]. Evidence suggests that maternal health care was trickling down to women in lower economic quintiles between 1996 and 2006 [16] before demand side financing was introduced. Improved primary school completion rate both among men and women, reduced proportion of people living below poverty line (except in far-west region), increased proportion of people living in urban areas, and expansion of maternity care services to Nepal's rural areas are likely to have contributed to these improvements.

Overall, greater attention is required to improve service availability and to target interventions to increase utilization of maternal health services to those in greatest need. Monitoring of inequality at the subnational level disaggregated by socioeconomic indicators is equally important to inform policy decisions. International experience reveals that services that are delivered through static health facilities and require access to tertiary care as in the case of delivery are likely to be more inequitable compared to interventions that are delivered at community level such as immunization and Vitamin A supplementation [34]. Experiences from Indonesia and Bangladesh show that free home-based care was propoor for delivery by trained service providers [35, 36]. While strong inequities in access to maternal care persist several promising initiatives are being tried by the Government of Nepal and have the potential to strengthen the health system deliver higher-level obstetric services more equitably, such as task shifting, incentivizing deployment of health workers to remote areas, and conditional cash transfers.

To increase equitable use of maternal health services in Nepal there is a need to strengthen the health system to raise utilization among poorer women, those with less education, and those living in remote areas. The high utilization of services by wealthy, urban, and educated women independent of other confounding factors indicates what can be achieved by improving the social status of women and development of rural areas. Nepal's more equitable family planning and immunization programs also point to what can be achieved in the country and suggest the need for better coordinated, integrated, and community outreach services for maternal health. Beyond the health sector, concerted efforts are needed to tackle the root causes of health inequity, improve economic status, improve female education, eradicate caste and ethnicity based social discrimination, and invest in the development of remote areas. The progressive approach to universal health coverage offered by Jamison and colleagues could be a promising policy action for Nepal in its commitment to ensure health as a basic human right by benefiting hard-to-reach sections the most [37].

The study has elicited important findings which could serve as a basis for ensuring equitable provision and utilization of maternal health services in Nepal. The large sample size by aggregating data from four NDHS surveys generates a high power for comparison of various population subgroups and yearly trend in health care utilization. However, the findings of the current study are subject to some limitations. First, the design of the four NDHS surveys may not have been strictly comparable although we tried to adjust some differences such as duration of recall period while assessing determinants of inequalities. Second, the findings are prone to recall bias since current analyses are cross-sectional and are based on data derived from recall of behaviour. Third, the perspectives of women who gave birth and died before an interview could take place were missed.

## 5. Conclusion

The findings of this study indicate that inequality in utilization of maternal health services in Nepal persists although it is in a declining trend from 1994 to 2011. Strategies encompassing both demand side and supply side interventions are required to address these inequalities. To turn the goals of universal health coverage into reality, special attention needs to be paid to poorer women, those with less education, and those living in remote areas. Obviously, policy and interventions also need to address development factors beyond the health system such as female education, women's economic empowerment, and the development of remote areas. Overall, it is essential that policies be structured and implemented to address context specific barriers if equality across population groups and regions is desired. Qualitative research on barriers to access, availability, and utilization of health care and other social services among poor, rural, and underserved populations is needed.

## Figures and Tables

**Table 1:** Number of households, women of reproductive age, and children under five by survey year.

	NFHS 1996	NDHS 2001	NDHS 2006	NDHS 2011
Total households (*n*)	8082	8602	8707	10826
Response rate (%)	99.6	99.6	99.6	99.4
Total women of reproductive age (15–49 yrs) (*n*)	8429	8726	10973	12674
Response rate (%)	98.2	98.2	98.4	98.1
Total married women of reproductive age (15–49 yrs) (*n*)	7978	8324	8244	9459
Total children under five (*n*)	4417	6931	5783	5306

**Table 2:** Maternal health services included in the study and their definitions.

	Definition/measurement
Four antenatal care check-ups (ANC)	Percentage of women aged 15–49 who had a live birth in the five years preceding the survey that received 4 or more antenatal check-ups

Institutional delivery	Percentage of live births in the five years preceding the survey delivered in a health facility (private or public)

Caesarian section delivery	Percentage of live births in the five years preceding the survey delivered by caesarian section in a health facility (private or public)

**Table 3:** Trend in sociodemographic characteristics of women who delivered in last five years^*∗*^ (1994–2011) and trends based on national household surveys.

	1994–1996 *N* = 4417	1997–2001 *N* = 6931	2002–2006 *N* = 5783	2007–2011 *N* = 5306	*P* value *(Chi-square test)*
*Ecological zone*					
Mountain	7.7	7.7	8.7	7.9	0.944
Hill	42.6	41.2	40.8	39.5	
Terai	49.7	51.2	50.5	52.6	
*Rural-urban residence*					
Urban	6.4	6.4	12.2	9.3	0.011
Rural	93.7	93.6	87.8	90.7	
*Education level*					
No education	79.3	74.2	60.3	47.3	<0.001
Primary	11.7	13.9	18.2	20.0	
Secondary	8.0	10.9	19.2	27.2	
Higher	1.0	1.0	2.4	5.5	
*Wealth quintiles*					
Poorest	25.9	25.4	25.5	25.8	0.996
Poorer	20.3	22.0	21.3	21.9	
Middle	20.2	20.0	20.4	21.0	
Richer	19.5	18.8	17.7	17.4	
Richest	14.1	13.7	15.1	13.9	
*Caste/ethnicity*					
Dalit	15.52	14.8	15.3	17.8	0.460
Brahmin/Chhetri	28.69	27.5	29.8	30.0	
Terai Madeshi Other Caste	12.69	17.0	13.0	10.4	
Newar	4.56	3.7	3.3	2.6	
Janajati	29.14	29.8	32.0	32.5	
Muslim	5.92	6.0	5.2	6.5	
Others	3.49	1.3	1.5	0.2	

^*∗*^Last 3 years for Nepal Family Health Survey 1996.

**Table 4:** Logistic regression analysis showing trends in maternity care indicators in Nepal (1994–2011), stratified by ecological zone, rural-urban residence, age group, wealth quintile education, and caste/ethnicity (*N* = 22,437).

	Four ANC		Institutional delivery		C-section	
	Univariate OR (95% CI)	Multivariate AOR (95% CI)	Univariate OR (95% CI)	Multivariate AOR (95% CI)	Univariate OR (95% CI)	Multivariate AOR (95% CI)
*Overall yearly trend*	1.18	1.18	1.16	1.17	1.14	1.12
	(1.15–1.20)	(1.15–1.19)	(1.14–1.18)	(1.15–1.19)	(1.11–1.18)	(1.09–1.15)
*Ecological belt*						
Mountain	1.23	1.25	1.22	1.21	1.136	1.05
	(1.17–1.29)	(1.19–1.32)	(1.16–1.28)	(1.14–1.27)	(1.02–1.25)	(0.91–1.19)
Hill	1.16	1.17	1.13	1.14	1.10	1.08
	(1.12–1.19)	(1.14–1.20)	(1.09–1.16)	(1.12–1.17)	(1.05–1.15)	(1.04–1.13)
Terai	1.19	1.18	1.19	1.19	1.18	1.15
	(1.16–1.22)	(1.15–1.20)	(1.16–1.22)	(1.16–1.22)	(1.13–1.23)	(1.11–1.20)
*Rural-urban residence*						
Urban	1.09	1.11	1.08	1.11	1.11	1.11
	(1.06–1.13)	(1.08–1.14)	(1.05–1.11)	(1.08–1.14)	(1.06–1.15)	(1.07–1.15)
Rural	1.19	1.19	1.19	1.18	1.16	1.13
	(1.17–1.22)	(1.17–1.21)	(1.16–1.21)	(1.16–1.21)	(1.12–1.20)	(1.09–1.17)
*Age group*						
15–19	1.15	1.15	1.20	1.23	1.09	1.10
	(1.12–1.19)	(1.11–1.19)	(1.16–1.24)	(1.18–1.28)	(1.02–1.18)	(1.01–1.19)
20–29	1.174	1.18	1.152	1.16	1.14	1.12
	(1.15–1.19)	(1.16–1.20)	(1.13–1.17)	(1.14–1.18)	(1.10–1.17)	(1.09–1.16)
30–39	1.19	1.18	1.19	1.19	1.21	1.16
	(1.16–1.22)	(1.15–1.21)	(1.15–1.22)	(1.16–1.23)	(1.13–1.29)	(1.09–1.24)
≥40	1.16	1.19	1.08	1.15	1.08	1.17
	(1.08–1.24)	(1.10–1.30)	(1.01–1.15)	(1.07–1.24)	(0.98–1.18)	(1.00–1.37)
*Wealth quintile*						
Poorest	1.23	1.22	1.17	1.15	1.16	1.13
	(1.19–1.27)	(1.18–1.26)	(1.13–1.22)	(1.11–1.19)	(1.04–1.27)	(1.02–1.25)
Poorer	1.23	1.210	1.214	1.181	1.05	1.03
	(1.19–1.28)	(1.170–1.251)	(1.174–1.254)	(1.141–1.222)	(0.942–1.171)	(0.91–1.16)
Middle	1.21	1.18	1.23	1.21	1.22	1.19
	(1.17–1.24)	(1.15–1.22)	(1.19–1.26)	(1.17–1.25)	(1.13–1.31)	(1.11–1.28)
Richer	1.22	1.18	1.25	1.19	1.18	1.15
	(1.18–1.25)	(1.15–1.21)	(1.21–1.28)	(1.16–1.22)	(1.11–1.25)	(1.08–1.23)
Richest	1.16	1.14	1.17	1.13	1.14	1.11
	(1.13–1.19)	(1.11–1.16)	(1.13–1.19)	(1.11–1.16)	(1.10–1.18)	(1.07–1.11)
*Education level*						
No education	1.15	1.18	1.16	1.18	1.10	1.13
	(1.13–1.18)	(1.16–1.20)	(1.13–1.19)	(1.15–1.21)	(1.05–1.15)	(1.07–1.19)
Primary	1.14	1.19	1.12	1.17	1.11	1.16
	(1.11–1.17)	(1.16–1.23)	(1.08–1.14)	(1.14–1.21)	(1.05–1.18)	(1.09–1.23)
Secondary	1.11	1.17	1.09	1.16	1.08	1.12
	(1.08–1.13)	(1.14–1.19)	(1.06–1.11)	(1.13–1.18)	(1.03–1.12)	(1.08–1.16)
Higher	1.12	1.19	1.06	1.14	1.07	1.12
	(1.07–1.18)	(1.13–1.26)	(1.01–1.11)	(1.08–1.21)	(1.01–1.14)	(1.05–1.19)
*Caste/ethnicity*						
Dalit	1.19	1.19	1.18	1.18	1.12	1.13
	(1.15–1.24)	(1.14–1.23)	(1.14–1.22)	(1.14–1.22)	(1.04–1.21)	(1.05–1.22)
Brahmin/Chettri	1.18	1.17	1.16	1.15	1.18	1.14
	(1.15–1.21)	(1.14–1.19)	(1.13–1.19)	(1.12–1.17)	(1.13–1.23)	(1.09–1.18)
Terai Madhesi Other Caste	1.17	1.15	1.20	1.19	1.15	1.12
	(1.12–1.22)	(1.11–1.19)	(1.14–1.26)	(1.13–1.25)	(1.06–1.25)	(1.04–1.21)
Newar	1.14	1.108	1.134	1.135	1.064	1.030
	(1.07-1.20)	(1.04-1.17)	(1.07-1.20)	(1.05-1.21)	(0.99-1.14)	(0.96-1.10)
Janajati	1.21	1.21	1.18	1.19	1.12	1.12
	(1.18-1.25)	(1.18-1.25)	(1.15-1.22)	(1.15-1.22)	(1.06-1.18)	(1.05-1.19)
Muslim	1.20	1.20	1.21	1.22	1.35	1.39
	(1.13-1.27)	(1.13-1.28)	(1.12-1.30)	(1.12-1.34)	(1.15-1.58)	(1.17-1.65)
Others	1.23	1.09	1.33	1.31	1.15	1.01
	(1.12-1.35)	(1.00-1.19)	(1.19-1.49)	(1.10-1.56)	(0.87-1.50)	(0.75-1.37)

**Table 5:** Sociodemographic predictors of four antenatal care visits, institutional delivery, and C-section in Nepal.

	Four ANC			Institutional delivery			C-section		
	(*N* = 13,211)			(*N* = 14,969)			(*N* = 14,969)		
	% use	Univariate OR (95% CI)	Multivariate OR (95% CI)	% use	Univariate OR (95% CI)	Multivariate OR (95% CI)	% use	Univariate OR (95% CI)	Multivariate OR (95% CI)
*Survey year*									
1994–1996	9.0	1	1	7.6	1	1	1.0	1	1
1999–2001	14.5	1.72(1.28–2.33)	1.81 (1.41–2.33)	9.8	1.33 (0.98–1.81)	1.33 (1.05–1.69)	1.0	1.00 (0.56–1.78)	0.99 (0.58–1.71)
2004–2006	30.3	4.42 (3.28–5.95)	4.20 (3.17–5.57)	19.1	2.89 (2.13–3.92)	2.51 (1.96–3.20)	3.0	3.07 (1.86–5.04)	2.67 (1.65–4.34)
2009–2011	51.6	10.84 (8.07–14.55)	11.53 (8.88–14.96)	40.6	8.38 (6.32–11.11)	9.80 (7.65–12.55)	5.3	5.55 (3.57–8.61)	4.83 (3.13–7.46)
*Ecological zone*									
Mountain	17.5	1	1	8.84	1	1	0.7	1	1
Hill	24.8	1.56 (1.15–2.11)	1.09 (0.84–1.41)	17.4	2.17 (1.56–3.00)	1.28 (0.96–1.72)	2.3	3.24 (1.88–5.58)	1.54 (0.90–2.66)
Terai	25.2	1.58 (1.17–2.14)	1.32 (0.97–1.78)	19.4	2.49 (1.80–3.43)	1.48 (1.11–1.98)	2.7	3.79 (2.26–6.38)	1.90 (1.07–3.38)
*Rural-urban residence*									
Urban	53.3	4.10 (3.31–5.07)	1.46 (1.19–1.79)	54.5	7.04 (5.70–8.70)	2.72 (2.23–3.31)	9.4	5.89 (4.26–8.15)	1.83 (1.29–2.59)
Rural	21.8	1	1	14.5	1	1	1.7	1	1
*Age group*									
15–19	28.5	6.67 (4.08–10.92)	2.99 (1.74–5.15)	22.9	4.51 (2.73–7.44)	1.83 (1.03–3.23)	2.5	1.26 (0.53–2.98)	0.46 (0.18–1.17)
20–29	28.1	6.56 (4.11–10.49)	2.77 (1.70–4.53)	19.9	3.76 (2.33–6.09)	1.31 (0.77–2.23)	2.5	1.28 (0.57–2.90)	0.35 (0.15–0.84)
30–39	15.5	3.08 (1.93–4.93)	1.88 (1.15–3.07)	11.3	1.93 (1.17–3.18)	1.01 (0.59–1.73)	1.9	0.97 (0.40–2.34)	0.40 (0.16–0.97)
≥40	5.6	1	1	6.2	1	1	2.0	1	1
*Education level*									
No education	11.5	1	1	7.9	1	1	1.0	1	1
Primary	32.1	3.64 (3.15–4.21)	2.06 (1.77–2.40)	20.4	3.02 (2.49–3.65)	1.74 (1.42–2.13)	2.5	2.58 (1.64–4.05)	1.41 (0.83–2.38)
Secondary	56.2	9.91 (8.35–11.77)	3.22 (2.71–3.82)	44.8	9.52 (7.89–11.48)	3.06 (2.50–3.73)	5.2	5.58 (3.87–8.05)	1.42 (0.84–2.40)
Higher	86.8	50.81 (34.49–74.87)	10.38 (6.81–15.81)	78.4	42.62 (29.80–60.95)	7.81 (5.08–11.99)	18.5	23.12 (14.48–36.92)	3.02 (1.63–5.60)
*Wealth quintile*									
Poorest	11.2	1	1	5.1	1	1	0.5	1	1
Poorer	16.8	1.60 (1.31–1.95)	1.50 (1.22–1.86)	10.2	2.09 (1.63–2.67)	1.82 (1.42–2.34)	0.5	1.11 (0.50–2.45)	1.02 (0.45–2.33)
Middle	22.4	2.28 (1.83–2.84)	2.03 (1.59–2.61)	15.4	3.37 (2.56–4.43)	2.66 (2.01–3.52)	1.6	3.38 (1.76–6.50)	2.87 (1.42–5.82)
Richer	29.6	3.33 (2.63–4.21)	2.64 (2.02–3.45)	21.7	5.10 (3.95–6.59)	3.57 (2.71–4.71)	2.8	5.83 (3.17–10.70)	4.22 (2.09–8.51)
Richest	54.5	9.46 (7.46–11.99)	5.08 (3.82–6.76)	50.0	18.45 (14.26–23.87)	9.00 (6.55–12.37)	9.0	20.09 (11.13–36.27)	9.37 (4.22–20.83)
*Caste/ethnicity*									
Dalit	18.5	1	1	12.4	1	1	1.0	1	1
Brahmin/Chhetri	33.6	2.23 (1.82–2.74)	1.48 (1.19–1.83)	23.6	2.19 (1.77–2.70)	1.24 (0.99–1.55)	3.8	3.78 (2.27–6.30)	1.85 (1.07–3.20)
Terai madhesi Other Caste	16.2	0.85 (0.64–1.14)	0.65 (0.49–0.87)	15.3	1.28 (0.92–1.78)	1.01 (0.76–1.33)	2.3	2.27 (1.23–4.17)	1.54 (0.83–2.88)
Newar	47.7	4.03 (2.79–5.82)	2.57 (1.76–3.77)	39.3	4.59 (3.18–6.62)	2.17 (1.51–3.13)	6.2	6.25 (3.44–11.38)	2.28 (1.25–4.15)
Janajati	21.9	1.24 (0.96–1.60)	0.93 (0.69–1.24)	14.2	1.18 (0.92–1.50)	0.85 (0.67–1.08)	1.6	1.56 (0.92–2.67)	1.12 (0.65–1.94)
Muslim	14.9	0.77 (0.50–1.18)	0.66 (0.47–0.93)	15.6	1.31 (0.73–2.32)	1.16 (0.71–1.92)	1.1	1.05 (0.32–3.45)	0.72 (0.24–2.16)
Others	13.9	0.71 (0.36–1.40)	0.92 (0.52–1.62)	13.7	1.13 (0.44–2.86)	1.477 (0.65–3.35)	1.0	0.94 (0.25–3.56)	0.73 (0.18–2.92)

## References

[1] Goland E., Hoa D. T. P., Målqvist M. (2012). Inequity in maternal health care utilization in Vietnam. *International Journal for Equity in Health*.

[2] Zere E., Tumusiime P., Walker O., Kirigia J., Mwikisa C., Mbeeli T. (2010). Inequities in utilization of maternal health interventions in Namibia: Implications for progress towards MDG 5 targets. *International Journal for Equity in Health*.

[3] Phiri J., Ataguba J. E. (2014). Inequalities in public health care delivery in Zambia. *International Journal for Equity in Health*.

[4] Zere E., Suehiro Y., Arifeen A., Moonesinghe L., Chanda S. K., Kirigia J. M. (2013). Equity in reproductive and maternal health services in Bangladesh. *International Journal for Equity in Health*.

[5] Zere E., Moeti M., Kirigia J., Mwase T., Kataika E. (2007). Equity in health and healthcare in Malawi: Analysis of trends. *BMC Public Health*.

[6] Zhao Y., You J., Wright J., Guthridge S. L., Lee A. H. (2013). Health inequity in the Northern Territory, Australia. *International Journal for Equity in Health*.

[7] Nepal Government, National Planning Commission (2013). Nepal millennium development goals. *Progress Report 2013*.

[8] Mehata S., Paudel Y. R., Mehta R., Dariang M., Poudel P., Barnett S. (2014). Unmet need for family planning in Nepal during the first two years postpartum. *BioMed Research International*.

[9] Khanal M. N., Shrestha D. R., Panta P. D., Mehata S. (2013). Impact of male migration on contraceptive use, unmet need and fertility in Nepal. *Further analysis of the 2011 Nepal Demographic and Heaalth Survey*.

[10] Mehata S., Baral S. C., Chand P. B., Singh D. R., Poudel P., Barnett S. (2013). *Nepal Household Survery*.

[11] Pandey J. P., Dhakal M. R., Karki S., Poudel P., Pradhan M. S. (2013). *Maternal and Child Health in Nepal: The Effects of Caste, Ethnicity, and Regional Identity*.

[12] Pradhan A., Suvedi B. K., Barnett S., Sharma S. K., Puri M., Poudel P. (2010). *Nepal Maternal Mortality and Morbidity Study 2008/2009*.

[13] Dim C. C., Ugwu E. O., Iloghalu E. I. (2013). Duration and determinants of inter-birth interval among women in Enugu, south-eastern Nigeria. *Journal of Obstetrics and Gynaecology*.

[14] Mehata S., Paudel Y. R., Dotel B. R., Singh D. R., Poudel P., Barnett S. (2014). Inequalities in the use of family planning in rural Nepal. *BioMed Research International*.

[15] Regmi K., Upreti S., Maureen D. I., Subedi H. N., Devi P., Kapil B. D. (2013). *A Study on Access to Maternal, Neonatal, and Child Health Services in Remote Areas of Nepal: Consolidated Report of Findings*.

[16] Johnson K., Bradley S. E. (2006). *Trends in economic differentials in population and health outcomes*.

[17] Commission on Social Determinants of Health (2011). *A Conceptual Framework for Action on the Social Determinants of Health*.

[18] Liu L., Johnson H., Cousens S. (2012). Global, regional and national causes of child mortality: an update systematic analysis for 2010 with time trends since 2000. *The Lancet*.

[19] https://mail.google.com/mail/u/0/#search/DHS/15c36b72aee73f6d

[20] Houweling T. A. J., Kunst A. E., Huisman M., Mackenbach J. P. (2007). Using relative and absolute measures for monitoring health inequalities: Experiences from cross-national analyses on maternal and child health. *International Journal for Equity in Health*.

[21] Moser K., Frost C., Leon D. A. (2007). Comparing health inequalities across time and place - Rate ratios and rate differences lead to different conclusions: Analysis of cross-sectional data from 22 countries 1991-2001. *International Journal of Epidemiology*.

[22] Wagstaff A., Paci P., van Doorslaer E. (1991). On the measurement of inequalities in health. *Social Science and Medicine*.

[23] Victora C. G., Matijasevich A., Silveira M., Santos I., Barros A. J., Barros F. C. (2010). Socio-economic and ethnic group inequities in antenatal care quality in the public and private sector in Brazil.. *Health policy and planning*.

[24] Chakraborty N., Islam M. A., Chowdhury R. I., Bari W., Akhter H. H. (2003). Determinants of the use of maternal health services in rural Bangladesh. *Health Promotion International*.

[25] Robson M., Hartigan L., Murphy M. (2013). Methods of achieving and maintaining an appropriate caesarean section rate. *Best Practice and Research: Clinical Obstetrics and Gynaecology*.

[26] Anwar I., Nababan H. Y., Mostari S., Rahman A., Khan J. A. M. (2015). Trends and inequities in use of maternal health care services in Bangladesh, 1991–2011. *PLoS ONE*.

[27] Family Health Division (2014). *Assessing Birthing Centres in Nepal*.

[28] CBS Nepal (2012). National population and housing census 2011. *National Report*.

[29] Family Health Division (2012). *Aama Program Guideline, Second Revision 2069*.

[30] Powell-Jackson T., Hanson K. (2012). Financial incentives for maternal health: Impact of a national programme in Nepal. *Journal of Health Economics*.

[31] Hodge A., Byrne A., Morgan A., Jimenez-Soto E. (2014). Utilisation of Health Services and Geography: Deconstructing Regional Differences in Barriers to Facility-Based Delivery in Nepal. *Maternal and Child Health Journal*.

[32] Agha S. (2011). Changes in the proportion of facility-based deliveries and related maternal health services among the poor in rural Jhang, Pakistan: Results from a demand-side financing intervention. *International Journal for Equity in Health*.

[33] Ir P., Horemans D., Souk N., Van Damme W. (2010). Using targeted vouchers and health equity funds to improve access to skilled birth attendants for poor women: A case study in three rural health districts in Cambodia. *BMC Pregnancy and Childbirth*.

[34] Barros A. J., Ronsmans C., Axelson H. (2012). Equity in maternal, newborn, and child health interventions in Countdown to 2015: A retrospective review of survey data from 54 countries. *The Lancet*.

[35] Hatt L., Stanton C., Makowiecka K., Adisasmita A., Achadi E., Ronsmans C. (2007). Did the strategy of skilled attendance at birth reach the poor in Indonesia?. *Bulletin of the World Health Organization*.

[36] Quayyum Z., Khan M. N. U., Quayyum T., Nasreen H. E., Chowdhury M., Ensor T. (2013). 'Can community level interventions have an impact on equity and utilization of maternal health care'—Evidence from Rural Bangladesh. *International Journal for Equity in Health*.

[37] Jamison D. T., Summers L. H., Alleyne G. (2013). Global health 2035: A world converging within a generation. *The Lancet*.

